# Exertional Heat Rhabdomyolysis in Class III Obesity Mimicking Nephrolithiasis and Hepatobiliary Disease

**DOI:** 10.7759/cureus.110493

**Published:** 2026-06-08

**Authors:** Carlos A Hernandez, Ayomide Sowemimo, Shanelle Brodeur, Obsient Merid

**Affiliations:** 1 Internal Medicine, Morehouse School Of Medicine, Atlanta, USA; 2 Internal Medicine, Morehouse School of Medicine, Atlanta, USA

**Keywords:** flank pain, heat stress disorders, myoglobinuria, nephrolithiasis, obesity, rhabdomyolysis

## Abstract

Exertional rhabdomyolysis has been described in military recruits and endurance athletes, whereas recognition in adults with severe obesity performing routine activities remains limited. Diagnostic delays may occur when initial clinical features resemble those of more common conditions and creatine kinase is omitted from early evaluation. A 30-year-old man with class III obesity (body mass index 51 kg/m²) developed severe right flank pain over three days after lawn mowing in hot weather. On presentation, he was febrile to 39.1°C, tachycardic, met systemic inflammatory response syndrome criteria, and had right costovertebral angle tenderness. Initial laboratory assessment demonstrated a 3+ heme urine dipstick, fewer than three red blood cells per high-power field, elevated aspartate aminotransferase (AST) and alanine aminotransferase (ALT), and normal alkaline phosphatase and bilirubin. Urologic and hepatobiliary evaluations were pursued, including computed tomography of the abdomen and pelvis and magnetic resonance cholangiopancreatography, whereas creatine kinase was not initially obtained. On admission the following morning, the presence of a dipstick-microscopy mismatch, AST-predominant transaminitis, recent heat exertion, and right thigh tenderness prompted the addition of creatine kinase to the diagnostic workup. Empirical isotonic crystalloid resuscitation was initiated before results were available. Creatine kinase was subsequently found to be 19,431 IU/L, and AST had declined from the emergency department value by the time of the result. Serum creatinine remained stable at 1.0 to 1.1 mg/dL, and the patient recovered without acute kidney injury. This case illustrates an atypical presentation of exertional rhabdomyolysis in which flank pain and transaminitis mimicked renal and hepatobiliary disease; recognizing the dipstick-microscopy mismatch and AST-predominant pattern after heat exertion should prompt clinicians to send creatine kinase and start fluids early.

## Introduction

Exertional rhabdomyolysis is a syndrome of skeletal muscle injury characterized by markedly elevated creatine kinase (CK), myoglobinuria, and the risk of acute kidney injury (AKI), electrolyte derangement, and, in severe cases, death. The contemporary diagnostic framework, established by the 276th ENMC (European Neuromuscular Centre) International Workshop, requires clinical features of muscle injury together with a CK above 10,000 IU/L for exertional cases and provides the RHABDO acronym to triage which patients warrant genetic evaluation [1]. The published epidemiology is dominated by military recruits and endurance athletes, with active-duty surveillance continuing to identify several thousand cases annually [2], and by patients with identifiable genetic substrates such as sickle cell trait [3]. The broader heat-related illness literature emphasizes deconditioning, inadequate hydration, and high ambient temperature as the principal triggers [4].

Less often illustrated is how the diagnosis is reached when the initial presentation is filtered through competing organ-system anchors before CK returns. Unilateral flank pain with a heme-positive urine dipstick directs the workup toward nephrolithiasis or pyelonephritis, and an AST-predominant transaminitis with preserved alkaline phosphatase and bilirubin directs it toward hepatobiliary disease or acute hepatitis, even when the underlying biochemistry points to skeletal muscle. Recognition matters because volume resuscitation given before the confirmatory CK is a key modifiable step in reducing AKI risk [5]. Obesity and unaccustomed exertion in heat are recognized substrates for exertional rhabdomyolysis, including in case reports of domestic labor such as lawn mowing, although the bedside recognition pattern receives less attention than the index event itself [4]. We describe a man in his thirties with class III obesity in whom an hour of lawn mowing in heat produced exertional rhabdomyolysis that initially triggered parallel urologic and hepatobiliary workups before the unifying diagnosis emerged.

## Case presentation

A 30-year-old man with class III obesity (BMI 51 kg/m²) and no significant past medical history presented to the emergency department with three days of severe right flank pain radiating to his groin. The pain began suddenly about one hour into mowing his lawn on a day averaging 82°F. The pain was initially rated 7 out of 10 and progressed to 10 out of 10 by the time he sought care, accompanied by one episode of non-bloody, non-bilious emesis and generalized weakness. He denied alcohol use, recreational or intravenous drug use, statin therapy, new medications, recent travel, prolonged immobilization, and any personal or family history of myopathy or exercise intolerance. On arrival, his temperature was 39.1°C, heart rate 120 beats per minute, and blood pressure 165/98 mmHg, with dry mucous membranes and marked right costovertebral angle tenderness. The combination of fever, flank pain, and a heme-positive urine dipstick anchored the emergency department differential on obstructing nephrolithiasis with superimposed pyelonephritis. Initial laboratory studies showed neutrophilic leukocytosis, normal creatinine, and a marked aspartate aminotransferase (AST)-predominant transaminitis with normal alkaline phosphatase and total bilirubin (Table 1); urinalysis was positive for blood on dipstick but contained few red blood cells (RBCs) on microscopy. CK was not ordered initially. Computed tomography of the abdomen and pelvis with contrast demonstrated punctate hyperdensity near the left ureterovesical junction (Figure 1), no other acute findings, and urology recommended no acute intervention. Right upper quadrant ultrasound showed hepatic steatosis with a mildly dilated common bile duct, prompting general surgery consultation and a recommendation for magnetic resonance cholangiopancreatography (MRCP).

**Figure 1:**
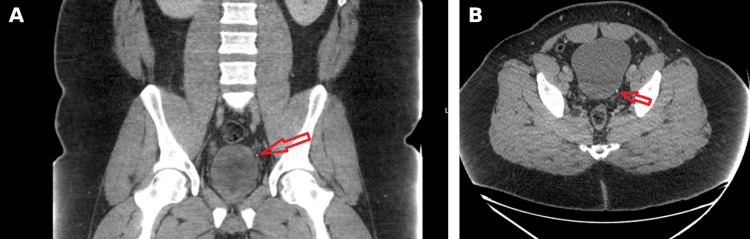
Computed Tomography of the Abdomen and Pelvis With Contrast (A) Coronal and (B) axial views demonstrating a punctate extraluminal calcification adjacent to the left ureterovesicular junction (red arrows). No right-sided calculus, no hydronephrosis, and no obstructing urolithiasis were identified.

**Table 1:** Initial Laboratory and Urinalysis Findings on Emergency Department Evaluation Emergency department orders included computed tomography of the abdomen and pelvis with contrast, right upper quadrant ultrasound, and general surgery consultation; creatine kinase was not part of the initial order set. (H) and (L) denote results above and below the reference range, respectively. Reference ranges are typical adult values and may vary by laboratory. AST: aspartate aminotransferase; ALT: alanine aminotransferase; HPF: high-power field.

Parameter	Value	Reference range	Units
White blood cell count	13.8 (H)	4.0–11.0	× 10⁹/L
Hemoglobin	12.9 (L)	13.5–17.5	g/dL
Creatinine	1.0	0.7–1.3	mg/dL
Blood urea nitrogen	8	7–20	mg/dL
AST	759 (H)	10–40	IU/L
ALT	229 (H)	7–56	IU/L
Alkaline phosphatase	47	44–147	IU/L
Total bilirubin	0.8	0.1–1.2	mg/dL
Albumin	3.8	3.5–5.0	g/dL
Lipase	5 (L)	13–60	IU/L
Potassium	3.5	3.5–5.0	mmol/L
Total calcium	8.5 (L)	8.6–10.2	mg/dL
Urine hemoglobin	3+	Negative	—
Urine red blood cells (microscopy)	<3/HPF	0–2/HPF	—
Creatine kinase	Not ordered	30–200	IU/L

The patient received a 1-liter lactated Ringer's bolus in the emergency department and was admitted to the inpatient medicine service in the morning. On examination, he had focal tenderness over the right anterior thigh without swelling, induration, or neurovascular compromise. CK was added to the workup, and an additional 1-liter bolus of normal saline was given, followed by a continuous infusion at 200 mL/hour, targeting a urine output of at least 1 to 2 mL/kg/hour. CK was markedly elevated (Table 2). Notably, AST had already declined from the emergency department value by the time the first CK was drawn. Creatinine remained within the reference range on presentation and during hospitalization (Tables 1, 2). The MRCP showed no choledocholithiasis, biliary dilation, or pancreatic abnormality, and viral hepatitis serologies were negative. 

**Table 2:** Trajectory of Laboratory Findings From Emergency Department Evaluation Through Discharge *First CK was drawn after morning reassessment on hospital day 1, at which point the patient had received one liter of lactated Ringer's in the emergency department and a subsequent one-liter bolus of normal saline ordered on reassessment; AST had already declined from the admission value before CK results were available. Continuous normal saline at 200 mL/hour was maintained thereafter. Dashes indicate values not measured on that draw. (H) and (L) denote results above and below the reference range, respectively. Reference ranges may vary by laboratory. CK: creatine kinase; AST: aspartate aminotransferase; ALT: alanine aminotransferase; ALP: alkaline phosphatase; LDH: lactate dehydrogenase; WBC: white blood cell count; ED: emergency department; DC: discharge.

Laboratory value	ED admission	Day 1 (AM)*	Day 1 (PM)	Day 2 (AM)	Day 2 (PM)	Day 4 (AM)	Day 4 (PM, DC)	Ref	Units
Creatine kinase	Not sent	19,431	13,085	12,272	6,205	3,448	2,923	30–200	IU/L
AST	759	475	377	329	174	83	64	10–40	IU/L
ALT	229	187	189	184	154	120	103	7–56	IU/L
Alkaline phosphatase	47	—	48	49	53	47	49	44–147	IU/L
Creatinine	1.0	1.1	1.0	0.9	0.9	0.9	0.9	0.7–1.3	mg/dL
LDH	—	525 (H)	—	—	—	—	—	140–280	IU/L
WBC	13.8 (H)	10.9 (H)	—	11.5 (H)	10.5	—	9.3	4.0–11.0	× 10⁹/L

## Discussion

This case illustrates that exertional heat rhabdomyolysis can present as unilateral flank pain with a heme-positive urinalysis and an AST-predominant transaminitis, a constellation that initially redirected the workup toward nephrolithiasis, pyelonephritis, and hepatobiliary disease before the unifying diagnosis emerged. The contemporary literature on exertional rhabdomyolysis is dominated by military recruits, endurance athletes, and patients with identifiable genetic substrates [1-3]. Less frequently discussed is the diagnostic pivot problem: recognizing exertional rhabdomyolysis in a community patient whose presentation, chief complaint, and initial laboratory pattern fit familiar renal and hepatobiliary frameworks, CK is not routinely ordered, and the diagnosis can be missed altogether rather than merely delayed. This case documents the specific biochemical and urinary patterns that should have led to earlier termination of the urologic and hepatobiliary workups.

Several findings in this case served as anchors, reinforcing the initial renal and hepatobiliary frameworks rather than redirecting the emergency department team toward muscle injury. The incidental punctate calcification near the left ureterovesical junction seen on computed tomography (Figure 1) had the radiographic appearance of a possible distal ureteral calculus, but on review was extraluminal and consistent with a phlebolith, which is a common venous calcification of no clinical consequence. In the context of right-sided flank pain, however, even a left-sided incidental finding can subtly support the working diagnosis of urolithiasis by suggesting a stone-forming substrate; this is the kind of finding that, if uncritically incorporated, prolongs the urologic workup despite the absence of any right-sided calculus or hydronephrosis. Similarly, the mildly dilated common bile duct and hepatic steatosis on right upper quadrant ultrasound obtained to explain the unexpected transaminitis were nonspecific and almost certainly preexisting, given the patient's body habitus, but their identification triggered a general surgery consultation and an MRCP. Neither imaging finding was incorrect; both were incidental, and both pulled the workup further from the actual diagnosis. Minimally low total calcium (8.5 mg/dL) occurred despite a normal albumin (3.8 g/dL), arguing against pseudohypocalcemia and instead suggesting early rhabdomyolysis, in which calcium deposits into injured muscle. Lipase was noncontributory. Atypical presentations of exertional rhabdomyolysis dominated by abdominal, flank, or back pain rather than myalgia are well described and are prone to misdirection toward gastrointestinal, urologic, or hepatobiliary diagnoses: periumbilical pain mimicking an intra-abdominal process [6], right upper quadrant pain initially attributed to cholecystitis [7], and abdominal wall injury mimicking an acute abdomen [8]. Our case adds heat-exertion-triggered unilateral flank pain with concurrent renal and hepatobiliary mimicry to this spectrum.

The diagnostic pivot in this case came not from a new test but from reexamining two findings already in the chart. A urine dipstick reacts to the heme moiety of both hemoglobin and myoglobin, and a strongly positive heme dipstick with few or no red blood cells on microscopy should raise suspicion for myoglobinuria, a point well established in the laboratory medicine literature on urinalysis interpretation [9]. The dipstick has poor positive predictive value for myoglobinuria on its own, and clinical context and CK remain necessary, but the dipstick-microscopy mismatch is a useful trigger to send CK [10]. The AST-predominant transaminitis with preserved alkaline phosphatase and bilirubin is similarly characteristic of skeletal muscle injury: skeletal muscle contains substantially greater AST activity than alanine aminotransferase (ALT) [11], and an AST-to-ALT ratio above one in an ambulatory patient without chronic liver disease, alcohol use, or hemolysis should prompt consideration of a muscle source. Each pattern alone is nonspecific. The diagnostic value in this case came from considering them together: a heme dipstick without true hematuria and transaminitis without cholestatic features, alongside focal anterior thigh tenderness on physical examination and a recent history of unaccustomed heat exertion.

The 276th ENMC International Workshop consensus by Kruijt and colleagues establishes the current diagnostic framework for rhabdomyolysis, defining exertional cases by clinical features of muscle injury together with a CK above 10,000 IU/L, and offers the RHABDO acronym to guide genetic testing [1]. Our patient met the exertional criteria on a clear heat-exertion trigger, with a rapid decline on supportive care, and lacked features that would invoke the RHABDO acronym: no recurrent episodes, no family history, no disproportionate symptoms relative to exertion, and no personal history of myalgia or exercise intolerance. The ENMC framework, therefore, validates conservative management without a neuromuscular workup, but it assumes the clinician has already reached the rhabdomyolysis differential. Our case illustrates the step before the ENMC algorithm engages: the recognition phase, in which the patient's chief complaint and initial laboratory pattern fit two more familiar syndromes, and the test that would confirm rhabdomyolysis had not yet been ordered.

The Kaiser Permanente cohort of hospitalized exertional rhabdomyolysis, as reported by Sabouri and colleagues, showed that NSAID (nonsteroidal anti-inflammatory drug) use and volume depletion, rather than absolute CK level, were primary drivers of AKI risk [5]. In contrast, the present patient had a markedly elevated CK, yet creatinine levels remained within the reference range throughout admission. This outcome is due to the absence of NSAID use, lack of chronic kidney disease, and prompt empirical crystalloid resuscitation started as soon as rhabdomyolysis was suspected. Intravenous fluids were given concurrently with the initial CK order, before laboratory results were available. The clinical significance of this intervention is seen in the laboratory trajectory. By the time CK results arrived, AST had already declined substantially from the emergency department value (Table 2). This decline is consistent with ongoing myoglobin clearance and reduced muscle injury during fluid administration. The decision to start fluids was guided by clinical pattern recognition rather than confirmed CK elevation. This early intervention aligns with, and may have contributed to, the absence of AKI. This case illustrates the findings of Sabouri et al. by showing that protective interventions, specifically early and adequate isotonic crystalloid administration with monitoring of urine output and electrolytes, and avoidance of nephrotoxins, must be implemented before confirmatory laboratory results. When CK is not included in the initial order set, fluid resuscitation should begin as soon as the clinical pattern is recognized.

The narrative review by Nguyen and colleagues catalogs ethnicity-linked and genetic susceptibility factors in exertional rhabdomyolysis, including sickle cell trait and inherited metabolic myopathies [3]. Our patient had no personal or family history suggestive of an inherited substrate, no recurrent episodes, and no disproportionate symptoms relative to the exertion encountered, which by the RHABDO criteria does not warrant genetic evaluation [1]. The trigger profile (class III obesity, deconditioning, unaccustomed exertion in summer heat) aligns with the heat-illness and obesity literature that identifies excess adiposity as an independent risk factor for exertional heat injury and rhabdomyolysis, including in occupational and recreational settings, and prior reports describe lawn mowing as a sufficient trigger in deconditioned adults [4].

The mechanistic basis for this association is multifactorial. Adipose tissue exhibits low thermal conductivity and serves as an insulating barrier to core-to-skin heat transfer. The extent of subcutaneous insulation increases with the degree of obesity, resulting in individuals with class III obesity dissipating a given metabolic heat load less efficiently than lean individuals performing the same task [12]. Severe obesity is independently associated with impaired body temperature regulation and a substantially higher rate of fatal heat stroke, characterized by both diminished heat-dissipation responses and reduced heat sensing [13]. In addition to this thermoregulatory disadvantage, higher absolute metabolic heat production is required to move a larger body mass during weight-bearing activity. Deconditioning and a tendency toward inadequate hydration further contribute to exertional heat injury, as recognized in the heat-illness literature [4]. Consequently, in a deconditioned adult with class III obesity, even an activity as routine as an hour of lawn mowing on a hot afternoon can generate a heat and muscle-injury burden that is disproportionate to the apparent intensity of the task.

This report has several limitations. It describes only one patient, reconstructs the cognitive pivot after the fact, and does not include genetic testing, which was not recommended by current guidelines [1]. Tests for acute viral hepatitis were negative, and the patient reported no alcohol use. Still, we cannot rule out a metabolic myopathy with low penetrance based on just one episode. We did not measure serum or urine myoglobin because our facility did not have the test available. Instead, we based the diagnosis on the mismatch between dipstick and RBC results and the CK trend, which is a common but less specific method [9]. The fluid management followed our usual institutional practice. It is still unclear whether balanced crystalloid is better than normal saline for exertional rhabdomyolysis. The patient's body type may have made it harder to assess volume status early on. The most useful counterfactual is the workup that was already underway. The MRCP had already been ordered when we reassessed the patient. If we had not noticed the dipstick-microscopy mismatch and the AST pattern at that time, the workup probably would have continued with more consultations, and CK might never have been checked.

## Conclusions

The presence of a heme-positive urine dipstick with few or no red blood cells on microscopy, along with AST-predominant transaminitis and preserved alkaline phosphatase and bilirubin, suggests skeletal muscle injury. This is especially true after recent heat exertion in deconditioned or obese individuals. Notably, CK is not routinely included in order sets for patients presenting with fever and flank pain. Recognizing this biochemical profile at the bedside may allow earlier consideration of skeletal muscle injury and reduce reliance on additional urologic or hepatobiliary diagnostic tests. Starting crystalloid resuscitation empirically, based on clinical pattern recognition rather than waiting for confirmatory CK measurement, may reduce the risk of AKI. This is the principal modifiable intervention in this clinical scenario.

## References

[1] Kruijt N, Laforet P, Vissing J, Bhai S, Stemmerik MG, Kleefeld F, Voermans NC (2025). 276th ENMC International Workshop: recommendations on optimal diagnostic pathway and management strategy for patients with acute rhabdomyolysis worldwide. 15th-17th March 2024, Hoofddorp, The Netherlands. Neuromuscul Disord.

[2] Armed Forces Health Surveillance Division (2025). Exertional rhabdomyolysis among active component members of the U.S. Armed Forces, 2020-2024. MSMR.

[3] Nguyen A, Thomassen A, MacDonald EM, Rodriguez VA, Brown JM, Shekoohi S, Kaye AD (2025). Ethnicity risk and exertional rhabdomyolysis: a narrative review. Adv Ther.

[4] Gauer RL MD, McNutt R MD, Bryan K MD (2026). Heat-related illnesses. Am Fam Physician.

[5] Sabouri AH, Yurgionas B, Khorasani S (2024). Acute kidney injury in hospitalized patients with exertional rhabdomyolysis. JAMA Netw Open.

[6] Alharbi KF, Alfahmi MZ (2023). Exercise-induced rhabdomyolysis manifestations and complications: a case report. Ann Med Surg (Lond).

[7] Haas DC, Bohnker BK (1999). "Abdominal crunch"-induced rhabdomyolysis presenting as right upper quadrant pain. Mil Med.

[8] Echague CG, Csokmay JM (2018). Exercise-induced abdominal wall muscle injury resulting in rhabdomyolysis and mimicking an acute abdomen. Obstet Gynecol.

[9] Simerville JA, Maxted WC, Pahira JJ (2005). Urinalysis: a comprehensive review. Am Fam Physician.

[10] Long B, Koyfman A, Gottlieb M (2019). An evidence-based narrative review of the emergency department evaluation and management of rhabdomyolysis. Am J Emerg Med.

[11] Nathwani RA, Pais S, Reynolds TB, Kaplowitz N (2005). Serum alanine aminotransferase in skeletal muscle diseases. Hepatology.

[12] Speakman JR (2018). Obesity and thermoregulation. Handb Clin Neurol.

[13] Kenny GP, Yardley J, Brown C, Sigal RJ, Jay O (2010). Heat stress in older individuals and patients with common chronic diseases. CMAJ.

